# Optimal interval of endoscopic screening based on stage distributions of detected gastric cancers

**DOI:** 10.1186/s12885-017-3710-x

**Published:** 2017-11-09

**Authors:** Chisato Hamashima, Rintaro Narisawa, Kazuei Ogoshi, Toshiyuki Kato, Kazutaka Fujita

**Affiliations:** 1 Division of Cancer Screening Assessment and Management, Center for Public Health Science, National Cancer Center, 5-1-1 Tsukiji Chuo-ku, Tokyo, 104-0045 Japan; 2 Division of Gastroenterology, Niigata Cancer Center Hospital, 2-15-3 Kawagishi-Cho Chuo-ku, Niigata, 951-8566 Japan; 3 Cancer Registry Section, Niigata Cancer Center Hospital, 2-15-3 Kawagishi-Cho Cyuo-ku, Niigata, 951-8566 Japan; 4 Committee of Gastrointestinal Cancer Screening, Niigata City Medical Association, 3-3-1 Shichikuyama Chuo-ku, Niigata, 950-0914 Japan

**Keywords:** Gastric cancer screening, Upper gastrointestinal endoscopy, Screening interval, Stage distribution, Survival rate

## Abstract

**Background:**

Although Korea and Japan have a national gastric cancer screening program, their screening intervals are different. The optimal screening interval of endoscopic screening in Japan was investigated based on the stage distributions of screen-detected gastric cancers.

**Methods:**

Patients with gastric cancer detected by endoscopic and radiographic screenings were selected from the Niigata City Medical Association database. The stage distributions of the detected gastric cancers were compared among patients with different screening histories in both groups. Gastric cancer specific survival rates were analyzed using the Kaplan-Meier method with the log-rank test.

**Results:**

There were 1585 and 462 subjects in the endoscopic and radiographic screening groups, respectively. In the endoscopic screening group, the stage IV proportion was lower in patients with screening history 1 and 2 years before diagnosis than in patients without screening history. Stage IV development was significantly related to the absence of screening history (*p* < 0.001); however, there were no differences between patients who had endoscopic screening history 2 and 3 years before diagnosis. The survival rates were not significantly different between patients with endoscopic screening 1 and 2 years previously (*p* = 0.7763). The survival rates were significantly higher in patients with endoscopic screening history 1 and 2 years before diagnosis than in patients without screening history (*p* < 0.001), and in patients with endoscopic screaming 3 years before diagnosis (*P* < 0.0069).

**Conclusion:**

The endoscopic screening interval for gastric cancer can be expanded to at least 2 years based on the stage distributions of detected cancers and the patient survival rates.

## Background

Gastric cancer is the third leading cause of cancer death all over the world. Despite the decrease in the incidence of gastric cancer in recent years, it still remains a heavy burden in eastern Asian and some European countries [1]. Although national gastric cancer screening programs have been effectively established in Asia, particularly in Korea and Japan, upper gastrointestinal endoscopic examination has already been performed as a standard examination for stomach diseases, and this procedure is also commonly used in the clinical setting worldwide [2]. In Korea, endoscopic screening for gastric cancer has been conducted since 1999 [3]. In Japan, endoscopic screening for gastric cancer as a national program was established only in 2016 based on the guidelines published by the National Cancer Center of Japan [4].

Before a new cancer screening technique is introduced in communities, the screening interval should first be defined in consideration of the balance of benefits and harms. Endoscopic screening is anticipated to have a high impact on mortality reduction of gastric cancer; however, it can cause serious harms including complications, false-positive cases, and overdiagnosis [5]. Screening programs should maximize mortality reduction in a defined screening interval for the given resources [6]. The Korean guidelines have defined the endoscopic screening interval as 2 years based on the results of a case-control study [7]. However, the Japanese guidelines have not clearly defined the gastric cancer screening interval based on conclusive evidence [8]. Moreover, as endoscopic screening resources are limited, its rapid dissemination remains difficult [9]. Therefore, the efficient use of resources should also be investigated to adequately disseminate and provide equal access to endoscopic screening for gastric cancer.

In Japan, Niigata City is considered to be the pioneer city for endoscopic screening since it was implemented there in 2003, much earlier than other municipalities [10, 11]. The total number of participants has now reached more than 45,000. Annual radiographic screening has also been provided in Niigata City.

In the present study, the optimal screening interval of endoscopic screening for gastric cancer was investigated according to the stage distributions of the screen-detected gastric cancers and the survival rates of patients with screen-detected gastric cancers using the Niigata City Medical Association database.

## Methods

### Screening programs

Annual gastric cancer screening using the upper gastrointestinal series has been started and provided by the local governments in Japan in accordance with the Health Service Law for the Aged since 1983 [12]. Since 2003, endoscopic examination has been added to the screening programs for gastric cancer in Niigata City [10, 11]. Both photofluorography and regular radiographic screening using the upper gastrointestinal series have been continued. Photofluorography has been performed as a mass screening program using mobile cars mainly in the suburbs of Japan. On the other hand, endoscopic and regular radiographic screenings have been performed in clinical settings in the center of cities. These screening programs have been basically performed for asymptomatic individuals. Individuals who visited regularly for disease treatment are often recommended to undergo cancer screening by their own primary care physicians. However, the underlying risk for the screened individuals in terms of developing gastric cancer was not clearly established. Individuals aged 40, 45, and 50 years or over can undergo endoscopic and regular radiographic screenings. Individuals aged more than 40 years can undergo photofluorography. Any screening method can be selected based on the individual’s preference. For all screening methods, there is no upper age limit and the screening interval is every year. Although the participation rate in gastric cancer screening has increased since the introduction of endoscopic screening, the screening rate has remained at approximately 25% [11].

The Niigata City Medical Association has provided endoscopic screening and regular radiographic screening to their member hospitals and clinics. Physicians who perform endoscopic screening for gastric cancer in Niigata City have been approved by the local committee for gastric cancer screening based on certain requirements [11]. Although these endoscopic screenings have been performed in clinical settings, the results have been evaluated by the local committee which included experienced endoscopists on the basis of a monitor screen review. In radiographic screening, a similar quality assurance system is used by the Niigata City Medical Association.

### Patients

The Niigata City Medical Association has developed a database for detected gastric cancer according to the screening method. The association has also systematically obtained detailed information related to gastric cancer from clinics and hospitals which have diagnosed and provided treatment for gastric cancer in Niigata City. The numbers of detected gastric cancer cases registered from 2003 to 2012 were 2420 for endoscopic screening and 572 for radiographic screening.

In the present study, the subjects were defined as individuals aged 40–79 years at the date of diagnosis of gastric cancer. All registered cases were ascertained by linkage with the Niigata Prefectural Cancer Registry. Even If there was no registration in the local cancer registry, cases with pathological information on the database of the Niigata City Medical Association were included. Patients with other cancers such as malignant lymphoma were excluded. The group was defined based on the screening method at the year of diagnosis. In the investigation of the screening interval for the same method, cases which had different screening histories of endoscopic and radiographic screenings were excluded.

### Statistical analysis

The basic characteristics of the gastric cancers detected by endoscopic and radiographic screenings were compared. Stage classification was based on the Japanese Classification of Gastric Carcinoma [13]. Gastric cancers were also classified histologically into intestinal and diffuse types according to Lauren’s criteria [14]. Differences in the proportion of both screening groups were compared using the chi-square test and student t-test.

The patients with gastric cancer detected by endoscopic and radiographic screenings were divided into 4 categories: (1) patients without screening history; (2) patients who had screening 1 year before diagnosis; (3) patients who had screening 2 years before diagnosis; (4) patients who had screening 3 years before diagnosis. Patients with no screening history were defined as those who had no screening history within 3 years before their diagnosis; however, patients who had screening history 4 or more years before their diagnosis were included. The cancer stage distributions by different screening histories in each screening group were compared using the chi-square test. Stage IV development was directly associated with gastric cancer death. The relationship between screening history and stage IV development was evaluated by logistic regression analysis.

Gastric cancer specific survival analysis of both screening groups with different screening histories was performed using the Kaplan-Meier method with the log-rank test. The obtained curves show the proportion of individuals alive over time from the time of screening. All test statistics were two-tailed, and *p*-values <0.05 were considered to indicate a statistically significant difference. Analyses were performed using STATA 13.0 (STATA, College Station, TX, USA).

This study was approved by the Institutional Review Board of the National Cancer Center of Japan.

## Results

### Subjects

**Fig. 1:**
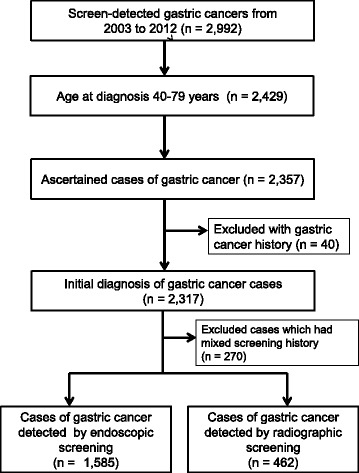
Flowchart of the selection process for the target group. A total of 2922 subjects were selected from the Niigata City Medical Association database, of which 563 patients were not within the target age for the analysis. Based on the ascertainment by local cancer registry or pathology reports which were collected from hospitals and clinics, 2357 patients were verified to have gastric cancer and 40 patients who had a history of gastric cancer before being registered as having cancer. To investigate the optimal screening interval in each screening, individuals who had different screening histories were excluded. Excluding those who had diffrent screening histories, the patients in whom gastric cancer was detected by gastric cancer screening were divided into 2 groups: endoscopic screening group (*n* = 1585) and radiographic screening group (*n* = 462)

The selection procedure for the target population is shown in Fig. 1. A total of 2922 subjects were selected from the Niigata City Medical Association database, of which 563 patients were not within the target age for the analysis. Most of the subjects who were excluded from the target group were more than 80 years old at the date of diagnosis, which was not the actual target age for cancer screening. On the basis of ascertainment using the local cancer registry or pathology reports which were collected from the hospitals and clinics, 2357 patients were verified as gastric cancer cases and 40 patients who had gastric cancer history before the cancer registration were excluded. To investigate the optimal interval for each screening, individuals who had different screening histories (mainly those who changed from radiographic screening to endoscopic screening) were excluded. Following the exclusion of patients with different screening histories, patients with detected gastric cancer were finally divided into 2 groups: endoscopic screening group (*n* = 1585) and radiographic screening group (*n* = 462).

The characteristics of the patients with gastric cancers detected by endoscopic and radiographic screenings were compared (Table 1). The age group and female ratio were not significantly different between the 2 groups. The distributions of screening histories for gastric cancer patients were nearly equal between both groups (*p* = 0.072). The cancer stage distributions were different between both groups, although 26.9% of the endoscopic screening-detected cancers lacked stage information. Half of the gastric cancers detected by endoscopic screening were treated by endoscopic submucosal dissection.

**Table 1:** Characteristics of gastric cancer patients and their gastric cancers detected by endoscopic and radiographic screenings

Screening method	Endoscopic screening		Radiographic screening		*p*-value
	Number	(%)	Number	(%)	
Total number	1585		462		
Sex					
Men	1097	69.2	322	69.7	0.842
Women	488	30.8	140	30.3	
Age group					
40–49 years	3	0.2	1	0.2	
50–59 years	69	4.4	20	4.3	0.647
60–69 years	614	38.7	164	35.5	
70–79 years	899	56.7	277	60.0	
Screening history^a^					
No screening history	688	43.4	169	36.6	
Screening 1 year before	715	45.1	231	50.0	0.072
Screening 2 years before	127	8.0	44	9.5	
Screening 3 years before	55	3.5	18	3.9	
Stage					
Stage I	944	59.6	284	61.5	
Stage II	66	4.2	45	9.7	
Stage III	48	3.0	32	6.9	< 0.001
Stage IV	50	3.2	31	6.7	
Unknown	427	26.9	70	15.2	
Pathology					
Intestine	1436	90.6	414	89.6	
Diffuse	115	7.3	34	7.4	0.719
Others	13	0.8	6	1.3	
Unknown	21	1.3	8	1.7	
Treatment					
Endoscopic submucosal dissection	793	50.0	127	27.5	
Surgery	703	44.4	293	63.4	< 0.001
Others	61	3.8	28	6.1	
Unknown	28	1.8	14	3.0	

^a^Screening history was limited to the same method of which gastric cancer was diagnosed

### Comparison of cancer stage distributions

The stage distributions of all detected gastric cancers between endoscopic and radiographic screenings were significantly different (*p* < 0.001). The stage distributions of gastric cancers detected by endoscopic screening between patients aged 40–69 years and 70 years and older were significantly different (*p* = 0.010). However, the stage distributions of gastric cancers detected by radiographic screening between patients aged 40–69 years and 70 years and older were not significantly different (*p* = 0.545).

**Fig. 2:**
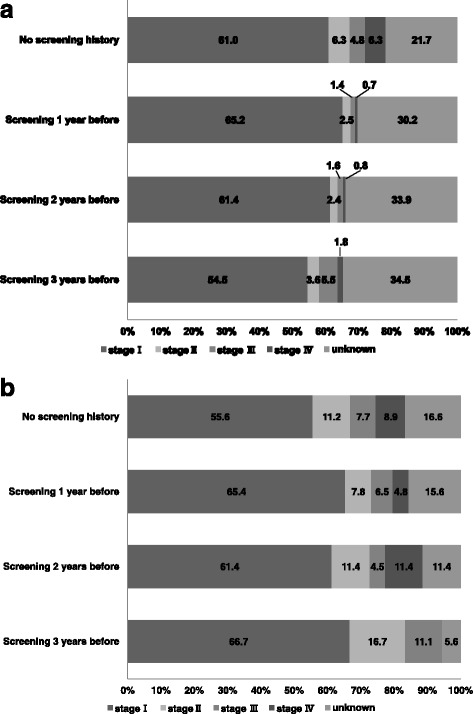
Stage distributions of gastric cancer among patients with different screening histories. Patients with gastric cancer detected by endoscopic and radiographic screenings were divided into 4 categories: (1) patients without screening history; (2) patients who had screening 1 year before diagnosis; (3) patients who had screening 2 years before diagnosis; (4) patients who had screening 3 years before diagnosis. Patients without screening history were included as well as individuals who had a screening history 4 or more years before diagnosis. Screening history was limited to the same method of which gastric cancer was diagnosed. **a** Stage distributions of gastric cancer detected by endoscopic screening. **b** Stage distributions of gastric cancer detected by radiographic screening

In endoscopic screening, the stage distribution of the detected gastric cancers was significantly different among the different screening histories (Fig. 2a, *p* < 0.001). More than 20% of the patients lacked cancer stage information in all the groups. In the first screening, the proportion of stage I was 61.0% and that of stage IV was 6.3%. The proportion of stage IV was not significantly different between patients who had endoscopic screening 1 year before diagnosis and patients who had endoscopic screening 2 years before diagnosis. In the patients who had endoscopic screening 3 years before diagnosis, the proportion of stage IV was 1.8%.

In radiographic screening, the stage distributions of the detected gastric cancers was not significantly different among the various screening histories (Fig. 2b, I = 0.463), and the proportion of stage I was 55.6% and that of stage IV was 8.9% in the patients without screening history. The proportion of stage IV was higher in patients who had radiographic screening 2 years before diagnosis than in patients without screening history.

In the patients with endoscopy-diagnosed cancer, although stage IV development was associated with no screening history (odds ratio = 9.575, *p* < 0.001), there were no differences between having screening history 2 and 3 years before diagnosis (Table 2). On the other hand, in the patients with radiographic screening-diagnosed cancer, any association with screening history could not be found.

**Table 2:** Odds ratios for stage IV development by logistic regression analysis

	Endoscopic screening			Radiographic screening		
	Odds ratio	95% CI	*p*-value	Odds ratio	95% CI	*p*-value
Sex						
Men	1			1		
Women	0.867	0.459–1.635	0.659	0.228	0.068–0.770	0.017
Age	1.004	0.959–1.051	0.875	1.004	0.940–1.072	0.904
Screening history						
Screening 1 year before	1			1		
Screening 2 years before	1.139	0.132–9.835	0.906	2.816	0.909–8.717	0.073
Screening 3 years before	2.613	0.300–22.773	0.385	–		
No screening history	9.575	3.739–24.517	< 0.001	2.164	0.942–4.971	0.069

^a^Screening history was limited to the same method of which gastric cancer was diagnosed

### Comparison of survival rates

As information regarding the stage of the detected gastric cancer was lacking, the survival rates among different screening histories were compared. The mean follow-up period was 67.6 ± 0.7 months (95% CI: 66.2–69.1). The 5-year survival rate of the patients with gastric cancer detected by endoscopic screening at 95.1 ± 0.5% (95%CI: 93.9–96.0) was significantly different from the 5-year survival rate of patients with gastric cancer detected by radiographic screening at 91.3 ± 0.1% (95% CI: 88.1–93.6) (*p* = 0.005). In patients without screening history, the survival rates were not significantly different between the endoscopic screening group and the radiographic screening group (*p* = 0.1331).

**Fig. 3:**
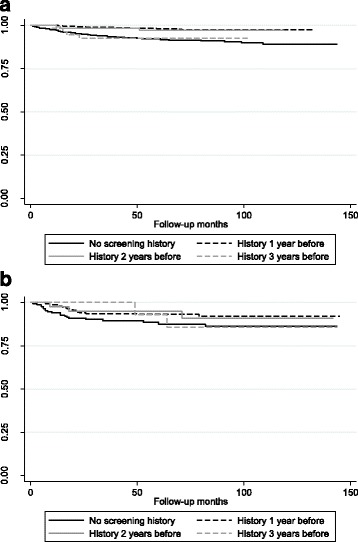
Survival rates of gastric cancer patients with different screening histories. Patients with gastric cancer detected by endoscopic and radiographic screenings were divided into 4 categories: (1) patients without screening history; (2) patients who had screening 1 year before diagnosis; (3) patients who had screening 2 years before diagnosis; (4) patients who had screening 3 years before diagnosis. Patients without screening history were included as well as individuals who had screening history 4 or more years before diagnosis. Screening history was limited to the same method of which gastric cancer was diagnosed. **a** Survival rates of gastric cancer detected by endoscopic screening. The 5-year survival rates among the 4 groups were follows: patients without screening history = 92.1 ± 0.1% (95% CI: 89.7–94.0); patients who had endoscopic screening 1 year before diagnosis = 98.1 ± 0.6% (95% CI: 96.6–98.9); patients who had endoscopic screening 2 years before diagnosis = 97.2 ± 1.6% (95% CI: 91.4–99.1); patients who had endoscopic screening 3 years before diagnosis = 92.6 ± 0.4% (95% CI: 81.4–97.2). **b** Survival rates of gastric cancer detected by radiographic screening. The 5-year survival rates in the 4 groups were follows: patients without screening history = 87.6 ± 2.7% (95% CI: 81.0–92.0); patients who had radiographic screening 1 year before diagnosis = 93.0 ± 1.7% (95% CI: 88.7–95.8); patients who had radiographic screening 2 years before diagnosis = 95.1 ± 3.4% (95%CI: 91.4–99.1); and patients who had radiographic screening 3 years before diagnosis = 92.9 ± 6.9% (95% CI: 59.1–99.0)

In the endoscopic screening group, the survival rates were significantly different among the 4 groups divided by screening history (Fig. 3a, *p* < 0.001). The 5-year survival rates among the 4 groups were follows: patients without screening history = 92.1 ± 0.1% (95% CI: 89.7–94.0); patients who had endoscopic screening 1 year before diagnosis = 98.1 ± 0.6% (95% CI: 96.6–98.9); patients who had endoscopic screening 2 years before diagnosis = 97.2 ± 1.6% (95% CI: 91.4–99.1); patients who had endoscopic screening 3 years before diagnosis = 92.6 ± 0.4% (95% CI: 81.4–97.2). The survival rates were not significantly different between patients with endoscopic screening 1 and 2 years previously (*p* = 0.7763). The survival rates were significantly higher in patients with endoscopic screening history 1 and 2 years before diagnosis than in patients without screening history (*p* < 0.001), and in patients with endoscopic screening 3 years before diagnosis (*P*<0.0069).

In the radiographic screening group, the survival rates differed but not significantly among the 4 groups divided by screening history (Fig. 3b, *p* = 0.2940). The 5-year survival rates in the 4 groups were follows: patients without screening history = 87.6 ± 2.7% (95% CI: 81.0–92.0); patients who had radiographic screening 1 year before diagnosis = 93.0 ± 1.7% (95% CI: 88.7–95.8); patients who had radiographic screening 2 years before diagnosis = 95.1 ± 3.4% (95% CI: 91.4–99.1); and patients who had radiographic screening 3 years before diagnosis = 92.9 ± 6.9% (95% CI: 59.1–99.0).

## Discussion

The effectiveness of endoscopic screening for gastric cancer has been increasingly reported in case-control and cohort studies in Korea, China, and Japan [15–18]. However, before introducing endoscopic screening in communities, the optimal screening interval must be defined to minimize harms and promote equal access to endoscopic screening for gastric cancer. Radiographic screening for gastric cancer has been performed since 1983 in Japan, and the screening interval has been defined annually [12]. In radiographic screening, the proportion of stage I was higher in patients who had screening 2 years before diagnosis than in patients who had no screening history. However, the survival rates of patients were similar regardless of the screening interval history among the patients whose gastric cancer was detected by radiographic screening. Based on the present results, the interval of radiographic screening cannot be easily expanded.

On the other hand, in endoscopic screening-detected cancers, the proportion of stage IV was lower in patients who had endoscopic screening 1 year or 2 years before diagnosis than in patients who had no screening history. The survival rates of patients who had endoscopic screening were also higher, and the rates were nearly equal between patients who had screening history 1 year versus 2 years before diagnosis. Stage IV development was strongly related to endoscopic screening history. The difference in the survival rates between the endoscopic and radiographic screening is caused by the significant difference in the stage distribution of the detected gastric cancer and the different treatment methods used by each screening methods. These results suggest that the interval of endoscopic screening for gastric cancer can be expanded to at least 2 years.

Previous studies conducted in Korea have concluded that the interval of endoscopic screening could be expanded to 2–3 years based on the stage distributions of the detected cancers and the previous endoscopic examinations [19–21]. In a nested case-control study conducted under a Korean national program, reduction in gastric cancer mortality could be confirmed even if the patients were screened only once within 4 years or more after the diagnosis of gastric cancer for case groups [16]. To define the screening interval, the natural history of gastric cancer should also be considered. In Eastern Asian countries, several studies and case reports have reported the natural history of gastric cancer based on a long-term follow-up [22–26]. Tsukuma et al. described the natural history of early-stage gastric cancer, and its mean duration of progression was 44 months from the early-stage to progress to the advanced stage [23]. The survival rates of patients with interval cancer of annual endoscopic screenings were nearly equal to the survival rates of patients with cancer detected by annual endoscopic screening [27]. These results indicate that it may be permissible to expand the endoscopic screening interval of gastric cancer from 1 year to 2 years or more.

The optimal interval for endoscopic screening of gastric cancer should be clearly defined to avoid unnecessary harms and decrease total number of examinations [28]. The value of cancer screening is determined by the difference of benefits and harms. As the intensity of cancer screening increases, the benefits of cancer screening rapidly increase. However, if the intensity of cancer screening increases beyond an optimal level, the harms and costs increase rapidly but not the benefits, decreasing the value of care [29]. Endoscopic screening also has harms such as false-negative results, false-positive results, compliments, development of infection, and overdiganosis [5]. As the upper age limit for endoscopic screening has not been defined in Japan, the target age should also be investigated to reduce harms and obtain the maximum screening effectiveness.

The present study has several limitations. *Firstly*, participation in radiographic screening has decreased nationwide in Japan, and this trend has also been observed in Niigata City. Therefore, patients with gastric cancer as detected by radiographic screening were limited in this study and the optimal screening interval could not be specifically defined for radiographic screening. *Secondly*, as the number of patients who had cancer screening 4 years or more before diagnosis was not sufficient, comparisons of the cancer stage distributions and patient survival rates were not adequate. *Thirdly*, the pathology of the detected cancers was mainly the intestinal type and their proportion was higher than the proportion of other types of detected cancers in other cities. Most of the patients in whom cancer was detected by gastric cancer screening were aged 60 years and above. In addition, data regarding a serious status appear to be lacking from the Niigata City Medical Association database. *Fourthly*, information regarding patients’ background and lifestyle was insufficient. Endoscopic examination has been commonly performed regardless of symptoms and has become commonly used as opportunistic screening. However, information as to whether or not the patients participated in opportunistic screenings remains lacking. People who are visiting their family physician have many opportunities to be screened. As these people have diseases that have been treated, they might constitute a high risk group for developing gastric cancer. However, this assumption could not be verified because of insufficient background information. *Finally*, information regarding *Helicobacter pylori* infection in the patients was unclear. Although the International Agency for Research on Cancer recommended the establishment of *H. pylori* screening and eradication programs in countries with a high incidence of gastric cancer, taking the local context into consideration is needed when it is introduced [30]. Notably, progression to gastric cancer differed according to the background of *H. pylori* infection and atrophy [31]. When risk assessment is combined with endoscopic screening, the screening interval may be further expanded for the low-risk group of gastric cancer. Further study is required on how to include risk assessment in gastric cancer screening.

## Conclusion

The interval of endoscopic screening for gastric cancer can be expanded to at least 2 years based on the stage distributions of the detected cancers and the patient survival rates. Further study regarding the optimal interval of endoscopic screening and the target age group in gastric cancer screening in Japan is warranted.

## Abbreviations

CI: Confidence interval
*H. pylori*: *Helicobacter pylori*
